# The microbial community and functional indicators response to flow restoration in gradient in a simulated water flume

**DOI:** 10.3389/fmicb.2022.1051375

**Published:** 2022-11-17

**Authors:** Wei Huang, Zhuowei Wang, Xiaobo Liu, Dayu Zhu, Yurong Wang, Leixiang Wu

**Affiliations:** ^1^State Key Laboratory of Simulation and Regulation of Water Cycle in River Basin, China Institute of Water Resources and Hydropower Research, Beijing, China; ^2^Department of Water Ecology and Environment, China Institute of Water Resources and Hydropower Research, Beijing, China; ^3^State Key Laboratory of Hydraulics and Mountain River Engineering, Sichuan University, Chengdu, Sichuan, China

**Keywords:** flow-reduced river reach, ecological response, eco-hydrology, flow restoration, microbial community

## Abstract

Flow reduction has greatly affected the river ecological systems, and it has attracted much attention. However, less attention has been paid to response to flow restoration, especially flow restoration in gradient. Flow regime of rivers may affect river functional indicators and microbial community structure. This study simulated the ecological restoration of the flow-reduced river reach by gradiently controlling the water flow and explores the ecological response of environmental functional indicators and microbial community structure to the water flow. The results showed that gross primary productivity (GPP), ecosystem respiration rate (ER) and some water quality indices such as chemical oxygen demand, total nitrogen, and total phosphorus (TP), exhibited positive ecological responses to flow restoration in gradient. GPP and ER increased by 600.1% and 500.2%, respectively. The alpha diversity indices of the microbial community increased significantly with a flow gradient restoration. Thereinto, Shannon, Simpson, Chao1, and Ace indices, respectively, increased by 16.4%, 5.6%, 8.6%, and 6.2%. Canonical correspondence analysis indicated that water flow, Dissolved oxygen and TP were the main influencing factors for changes in bacterial community structure. Microbial community structure and composition present a positive ecological response to flow restoration in gradient. This study reveals that the main variable in the restoration of the flow-reduced river reach is the flow discharge, and it provides a feasible scheme for its ecological restoration.

## Introduction

Human activities such as dam construction, water diversion and water transfer, have resulted in a substantial reduction (over 80.0%) of the flow of some river sections or even drying, forming flow-reduced river reach in the lower reach of many hydroelectric projects globally (Chen and Olden, 2017; Grill et al., 2019). Previous studies have shown that the hydrological regime of the flow-reduced reaches have changed significantly, resulting in serious degradation of the structure and function of the river ecosystem (Thieme et al., 2020; Zahedi et al., 2021, 2022). Tonkin et al. (2018) found that maintaining water flow regime would overcome the negative consequences of flow alteration on river ecosystems. Therefore, the ecological problem of the flow-reduced river reach has attracted extensive attention from all over the world. Scientifically restoring the environmental flow of the flow-reduced river reach and maintaining the integrity of the ecosystems in the flow-reduced reach have been an urgent problem that needs to be solved. Poff and Schmidt (2016) pointed out that the ecological and social functions of some degraded rivers can be restored to a certain extent by adjusting the discharge flow. However, the mechanistic relationship between water reduction and ecological microbial structure changes remains unclear. The ecological response of hydrological regime restoration is still unclear, resulting in a lack of theoretical basis for ecological flow restoration.

Water resources departments and researchers globally have conducted a lot of monitoring and research on the ecological responses of algae and aquatic plant populations and environmental functional indicators in the lower reach of the flow-reduced river. Dewson et al. (2007) carried out *in-situ* water reduction experiments, and found that there was a significant relationship between the retention rate of organic matter and flow. Flinders and Hart (2009) studied the response of harmful attached algae to pulse floods through field tank experiments and obtained a quantitative relationship between flow changes and attached harmful algal biomass. James and Suren (2010) and Song et al. (2022) studied the response of the microbial community to the reduction of flow gradient and found that flow was the key factor affecting the biomass of the microbial community. It can be seen the water flow in the flow-reduced river reach may be one of the main indicators affecting the restoration of the microbial community. However, current research mainly focuses on the response or ecological effects of river reaches to flow reduction. Few studies have been reported on experiments on the response of flow-reduced river reach to flow restoration.

Microbial communities in aquatic ecosystems are exceedingly sensitive to changes in the living environment (Moitra and Leff, 2015; Falfushynska et al., 2019). Alteration of microbial communities can response alteration of ecosystem structure and function (Woodhouse et al., 2016; Cai et al., 2017). Flow regime of rivers may affect river functional indicators and microbial community structure. These functional indicators are important indicators to indicate the healthy and operation of the ecosystem, such as gross primary productivity (GPP) and ecosystem respiration (ER). Therefore, in this study, the actual river water was diverted into the simulated flume, and a flow gradient restoration was simulated by controlling the flow rate of each section. That is used to explore the impact of flow restoration on environmental functional indicators and microbial community structure in ecosystems. The aims are to (1) reveal the ecological response of bacterial community structure and environmental functional indicators with a flow gradient restoration (2) explore the feasibility of the ecological restoration of flow-reduced river reach by controlling water flow. Furthermore, it provides a better solution for ecological restoration of the flow-reduced river reach.

## Materials and methods

### Sampling location and simulation process

**Figure 1:**
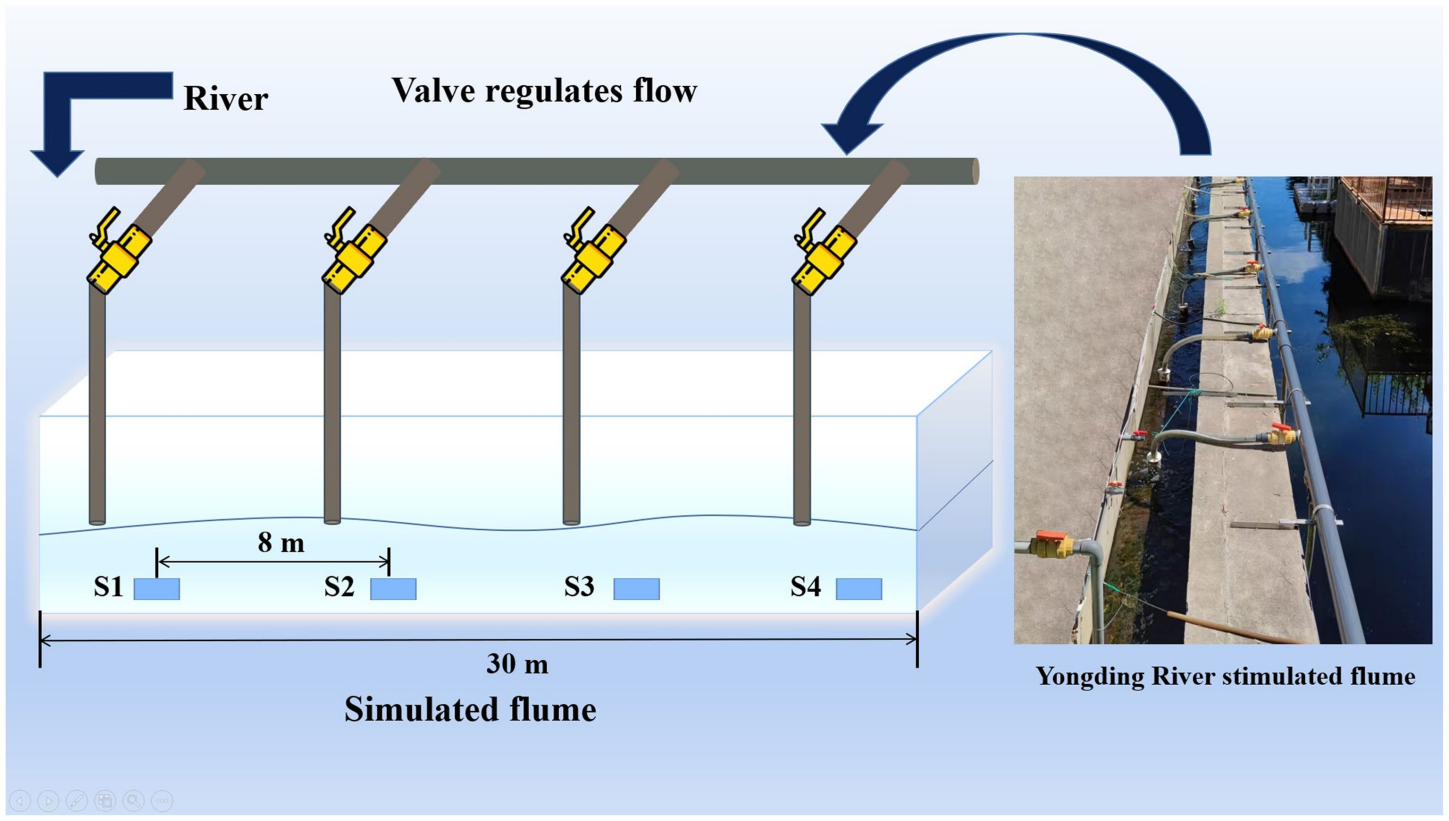
Process diagram of simulated water flume experiment.

This study was conducted downstream of the Yongding River Small Hydropower Station in Yuyuan, Beijing, China. A 30 meters long simulated water flume was constructed nearby (Figure 1). Eight water pumps were placed at the bottom of the Yongding River to pump water above the simulated flume. The flow velocity (Fv) is adjusted to control the flow regime, by the same cross-sectional area of the flume. The Fv from S1 to S4 is increasing in gradient (The Fv information is shown in Table 1). The water flow into each section (four different flow velocities) of the simulation flume was controlled by four valves. S1–S4 represented sampling points for four different gradient flow stages in the simulated flume.

**Table 1:** Water quality index after the restoration of flow in gradient.

Water quality index	S1	S2	S3	S4
Fv (m/s)	0.072 ± 0.003	0.087 ± 0.013	0.142 ± 0.003	0.186 ± 0.003
pH	7.524 ± 0.013	7.596 ± 0.002	7.595 ± 0.098	7.601 ± 0.024
DO (mg/L)	10.42 ± 0.98	10.37 ± 1.12	10.38 ± 1.45	10.380 ± 0.78
TDS (mg/L)	948 ± 13	947 ± 20	947 ± 6	947 ± 11
ρ (Ω·cm)	1,056 ± 1	1,056	1,056 ± 2	1,056
TN (mg/L)	12.4 ± 0.12	13.9 ± 0.23	12.7 ± 0.98	12.2 ± 2.13
TP (mg/L)	0.1 ± 0.01	0.09	0.08 ± 0.02	0.09 ± 0.01
TS (mg/L)	24 ± 2	23 ± 2	11 ± 2	14 ± 4
NO_3_^−^ (mg/L)	6.7 ± 0.3	7 ± 0.5	6.6 ± 0.2	6.2 ± 0.5
NO_2_^−^ (mg/L)	0.014 ± 0.004	0.011 ± 0.004	0.01 ± 0.001	0.01 ± 0.003
NH₄^+^ (mg/L)	0.13 ± 0.001	0.13 ± 0.023	0.13 ± 0.019	0.12 ± 0.014
COD (mg/L)	28 ± 3	34 ± 2	24 ± 3	26 ± 2
Chla (SPAD)	11.679	12.33	15.334	20.374

The values are expressed as the means ± SD (*n* = 3).

### Samples collection

Water samples were collected from each sample point with a water collector and collected in 150 ml polyethylene bottles. The water samples were then filtered through Whatman GF/F filters (0.22 μm pore size), stored at <4°C (without freezing) and physicochemical parameters were determined within 24 h.

### Environmental function indicators analysis

Chlorophyll-a (Chla) testing from germinating algae was collected on artificial substrates with S1–S4. Four groups of artificial substrates were placed in two rows in the central area of each experimental interval and marked. One group of artificial substrates was randomly selected, and 10 cm^2^ of borne algae were scraped successively with a toothbrush in each group of artificial substrates, rinsed with distilled water into 500 ml wide-mouth plastic bottles, and brought back to the laboratory for testing. The above indicators were analyzed by Quasi-Dual Beam UV–Vis Spectrophotometer (DR6000, Hach Co., Ltd., USA). The single-station open-channel oxygen method was used for characterizing GPP and ER (Hall and Hotchkiss, 2017).

### Physicochemical analyses

pH, dissolved oxygen (DO), total dissolved solids (TDS), water resistivity (ρ) and other parameters were measured on-site using the Multi 3,630 IDS (Xylem inc., Germany) portable multi-parameter meter. Samples for total phosphorus (TP), total nitrogen (TN), total sulfur (TS), permanganate index (COD_Mn_), nitrate (NO_3_^−^), nitrite (NO_2_^−^), ammonia nitrogen (NH₄^+^) and chlorophyll a (Chla) were analyzed by the China Ministry of Water Resources Water Quality Supervision, Inspection and Testing Center. Fv was monitored using a Portable Flow Calculator (LS002280, Nanjing Xiangruide Electric Technology Co., Ltd., China).

### DNA extraction and sequencing data analysis

The 1 l water sample was collected by a water collector from the downstream to the upstream. The water samples below the water surface 5 cm were collected in sequence, and the water samples were brought back to the laboratory for 30 min (to reduce the interference of sediments and algal biofilm microorganisms on the samples). The four water samples were suction filtered (0.22 μm glass fiber membrane), and the filter membranes were the microbial community samples. It was transported to Beijing Nuohezhiyuan Technology Co., Ltd. in ice packs. DNA was extracted using the CTAB method (Hultman et al., 2015). The primers 515F (CCTAYGGGRBGCASCAG) and 806R (GGACTACNNGGGTATCTAAT) were used to amplify the V3–V4 region of the bacterial 16S rRNA gene. High-throughput sequencing analysis of the bacterial rRNA genes was performed using Novomagic, a free online platform for data analysis platform^1^. The entire Effective Tags of all samples are clustered using the Uparse algorithm (Uparse v7.0.1001^2^), and by default the sequences are clustered with 97.0% consistency (Identity) into OTUs (Operational Taxonomic Units), while a representative sequence of OTUs will be selected, based on the principle of its algorithm, the sequence with the highest frequency of occurrence in OTUs is selected as the representative sequence of OTUs. Species annotation of OTUs sequences was analyzed by the Mothur method with the SSUrRNA database from SILVA138^3^ for species annotation (setting a threshold of 0.8 to 1) to obtain taxonomic information and separately at each taxonomic level: kingdom (boundary), phylum, class, order, family, genus, species, and the community composition of each sample. Rapid multiple sequence matching was performed using MUSCLE (Version 3.8.31^4^) software to obtain the phylogenetic relationships of all OTUs representative sequences. Finally, the data of each sample were homogenized, and the least amount of data in the sample was used as the criterion for homogenization, and the subsequent Alpha diversity analysis and Beta diversity analysis were based on the homogenized data.

### Statistical analysis

This study uses water flow as the main variable to explore the ecological responses of other environmental factors. The response indicators screened in this study were GPP and ER. These water quality indexes were analyzed by Spearman correlation. Canonical correspondence analysis (CCA) was performed using the vegan package to identify environmental variables mainly associated with changes in bacterial community structure. Louca et al. (2016) classified the ecological functions of bacteria and archaea in the environment by using the published literature evidence and then compiled the FARPOTAX database. Based on the annotation results of amplicon species, researchers can map prokaryote branches (such as genera or species) to establish metabolism or other ecological-related functions. α-diversity was estimated with the community richness indices (Ace, Chao), the community diversity indices (Shannon, Simpson), Good coverage and Sequence number. The data from this study are statistically analyzed and plotted by Origin 2021 pro.

## Results

### Response of functional indicators and water quality indexes to alter flow in gradient

**Figure 2:**
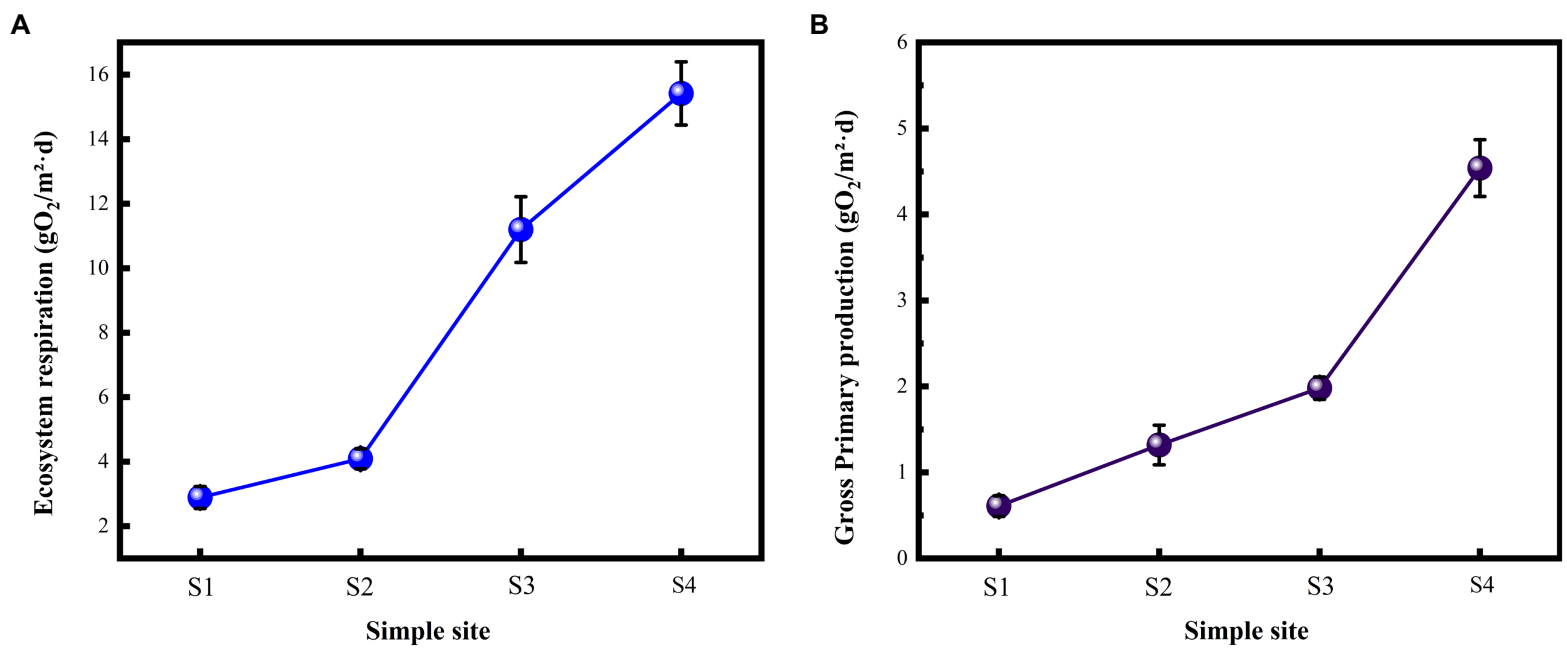
Responses of river metabolism indicators with flow changes. **(A)** Ecosystem respiration; **(B)** Gross primary production.

The environmental functional indicators of this study were selected as the metabolic indicators of the river, GPP and ER (Figure 2). GPP and ER showed a gradual upward trend with the gradient restoration of water flow. The GPP and ER of S4 sampling point are 6.98 and 5.15 times that of S1, respectively.

**Figure 3:**
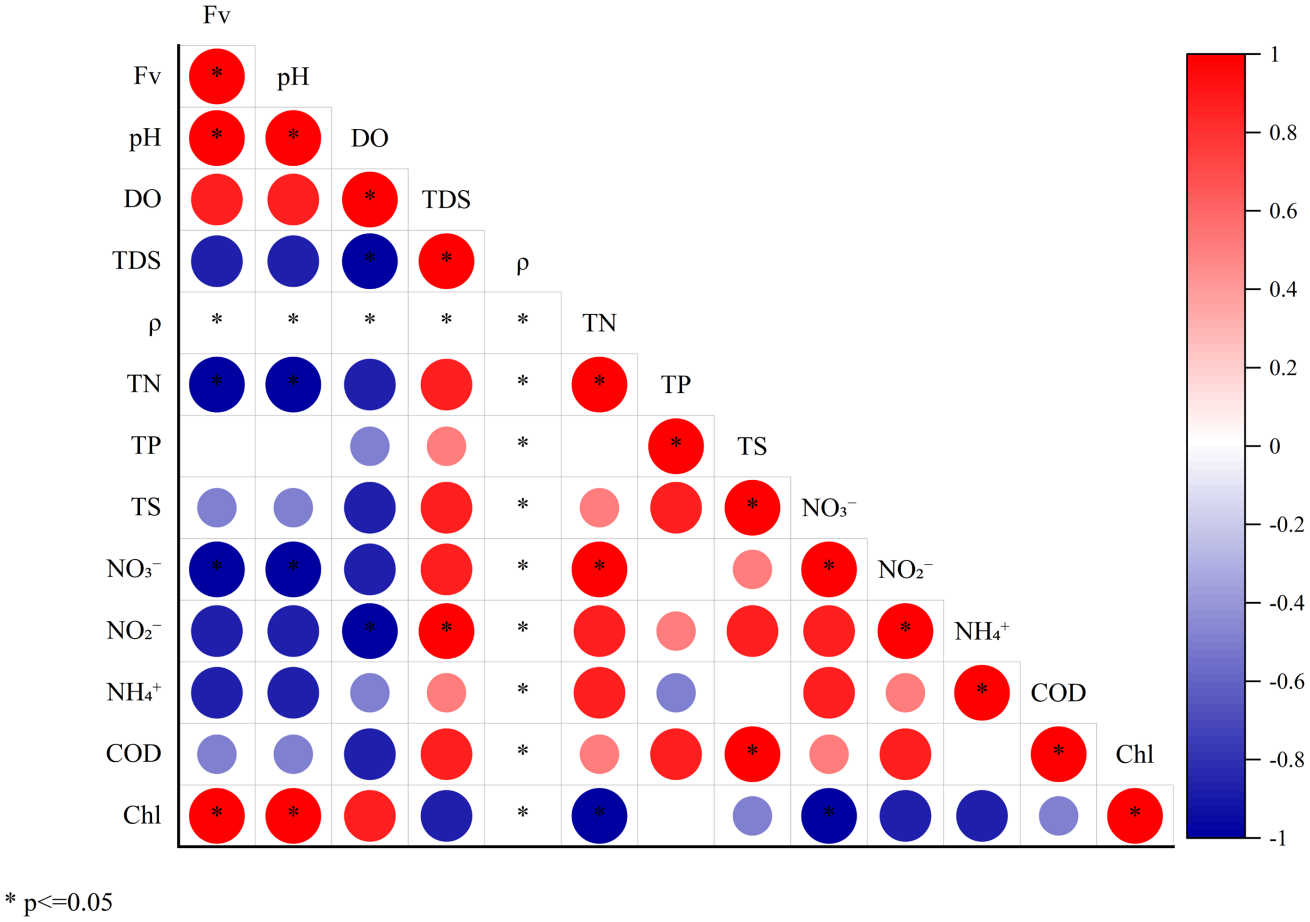
Spearman correlation heat map of environmental functional factors and Fv (* means the significant correlation between the two indicators, *p* < 0.05).

The characteristics of water quality indexes and Spearman correlation analysis of the four sampling points with increasing Fv gradient in the simulated flume are shown in Table 1 and Figure 3. The TDS and ρ of the four sampling points remained the same with the Fv of the gradient increasing. With the increase of flow gradient, the two environmental function indicators of pH and Chla showed a significant positive linear correlation (Spearman analysis, *p* < 0.05). However, NO_3_^−^ concentration showed a significant negative liner correlation (Spearman analysis, *p* < 0.05). In addition, TN, TP, TS, NO_2_^−^, NH₄^+^ and COD showed a certain negative correlation with water flow. The results of the comprehensive environmental water quality index show that with flow gradient restoration, the indicators in the simulated flume showed relatively positive ecological response, gradually improving the water quality (Mu et al., 2020).

### Response of microbial community structure, richness, and diversity to gradient flow

**Figure 4:**
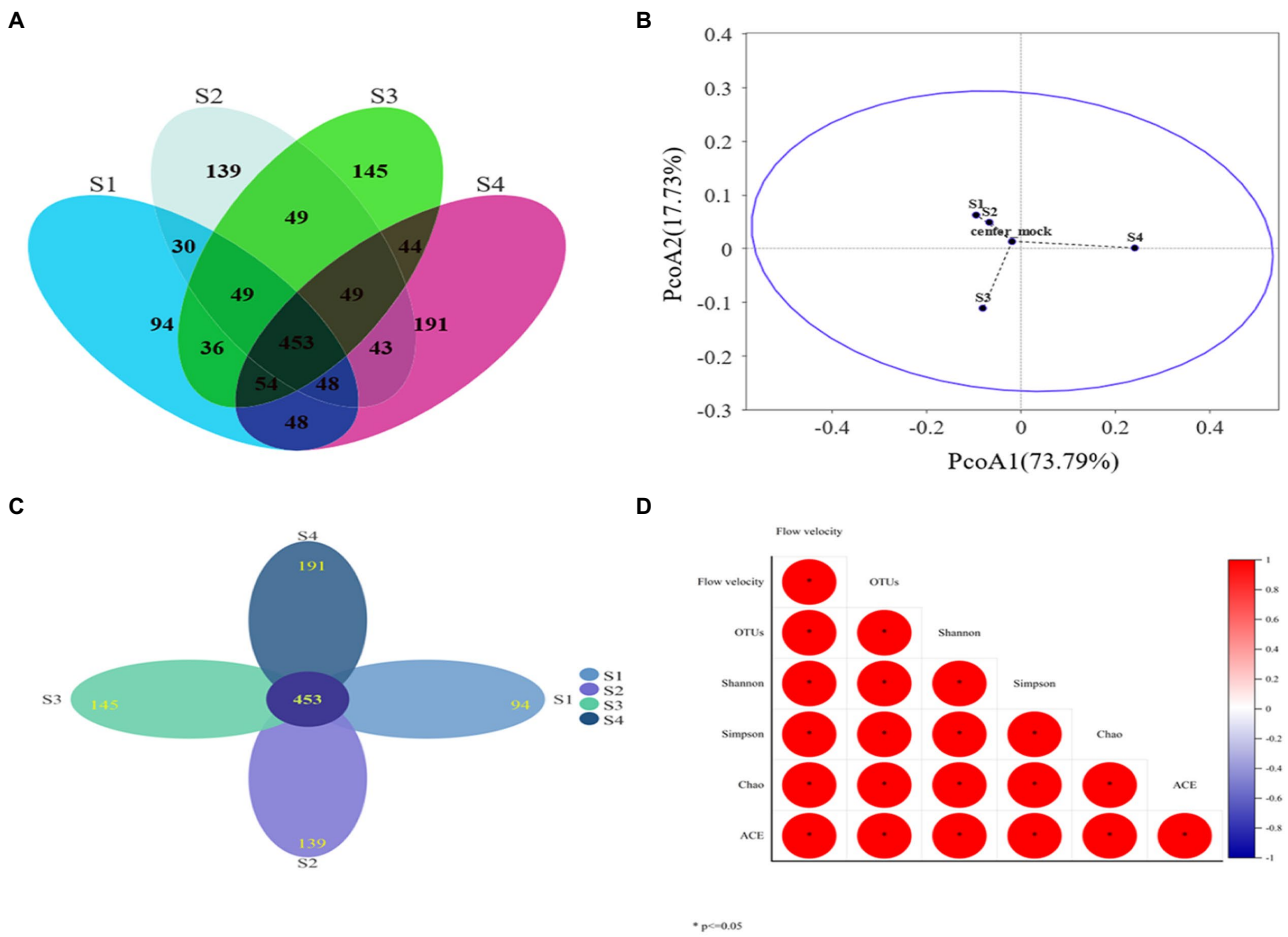
**(A)** Venn diagram of common and unique OTUs present in microbial communities under different water flow conditions; **(B)** PcoA analysis of OTUs (distance algorithm: Bray-Curtis); **(C)** Flower figure, each petal represents a sample, different colors represent different samples, the core number in the middle represents the number of OTUs common to all samples, and the number on the petal represents the number of OTUs unique to the sample. **(D)** Spearman correlation heat map of Alpha diversity index and flow velocity (* means significant correlation between the two indicators, *p* < 0.05).

After chimera removal and filtering, a total of 264,780 sequences were obtained from Illumina NovaSeq sequencing. The sequencing depth coverage of each sample exceeded 99.5%, proving that 99.5% of the microbial species in the four samples had been identified. The results showed that the coverage was sufficient to support the subsequent analysis of microbial community structure (OTU cluster, Alpha diversity analysis, etc.) (Zhu et al., 2022). The Alpha diversity index was listed in Table 2 and the number of OTUs in Figure 4A. The Spearman analysis of Figure 4D intuitively shows that, with the gradient of water flow, the microbial diversity indices, including community richness indices (Ace and Chao), community diversity indices (Shannon and Simpson), and the number of OTUs, showed significant positive liner correlation (Spearman analysis, *p* < 0.05). Specifically, when the flow gradient was restored to the S4 stage, the community richness, diversity and the number of OTUs of the microbial community were the largest, the Chao index increased from 915.829 to 994.853, the Shannon index increased from 0.887 to 0.945 and the number of OTUs increased from 812 to 909. In addition, the difference of OTUs levels is shown in Figure 4A, with 1952 OTUs clustered in four different water flow samples and 94,139,145,191 unique OTUs belonging to S1, S2, S3, and S4 samples, respectively. Core 453 OTUs (23.5% of total OTUs) were shared by these microbes in these sampling sites (Figure 4C). The species composition structure of the four samples was analyzed by PcoA (Figure 4B). The close distance between S1 and S2 (Group 1) indicates that the species composition structure of the two was relatively similar. However, while S3, S4, and S1 by Bray-Curtis distance algorithm showed that the three distances were far away, so 3 microbial communities (Group 1, S3, and S4) were quite distinguished. This indicated that there were large differences among the four samples, further supporting the validity of the richness and diversity of the microbial community. The results showed that the indicators of the microbial community structure performed excellent ecological response to a gradient restoration of the water flow, and the richness and diversity of the community were restored steadily.

**Table 2:** Alpha diversity indexes of microbial community in each sampling site.

Sample	Sequence number	Shannon	Simpson	Chao	Ace	Goods coverage
S1	71,043	5.204	0.887	915.829	952.982	0.995
S2	61,824	5.377	0.891	926.667	963.349	0.995
S3	72,531	5.464	0.893	941.568	988.044	0.995
S4	59,382	6.057	0.945	994.853	1,011.868	0.995

Phylogenetic classification results showed 44 bacterial phyla in total samples, including 59 classes, 129 orders, 221 families and 369 genera. The bacterial community composition at the phylum level in samples was shown in Supplementary Figure S1. Overall, the bacterial communities in the simulated flume were dominated by *Proteobacteria*, *Firmicutes*, and *Bacteroidetes* which were widespread in other freshwater rivers (Mou et al., 2013; Zemskaya et al., 2022). In our study, these three phyla remain stable with mean relative abundances of 78.0%, 6.4%, and 3.8%, respectively. In contrast, with the restoration of the gradient of water flow, the relative abundance of *Cyanobacteria* showed a significant decrease from 8.06% in S1 to 1.0% in S4 gradually. However, the relative abundance of other phyla (except for the relative abundance of TOP 10) gradually increased from 2.0% to 5.3%, indicating that the restoration of water flow promotes the gradual increase of the number of phyla and the diversity of microbial community species.

At the genus level, the bacterial community structure has been analyzed in more detail using a heat map of the top 40 genera. To better characterize the response of microbial communities from different water flow samples. As shown in Supplementary Figure S2, in the S1–S4 bacterial groups the dominant genera were *Undibacterium* and *Acidovorax*, with an average relative abundance of 22.4 and 19.1%, respectively. However, the relative abundances of *Undibacterium* and *Acidovorax* in S4 were 12.2% and 17.6% with flow restoration in gradient. In addition, the genus clustering heat map showed that emerging clustering bacteria genera appeared in the S4 samples, such as *Enterococcus*, *Bdellovibrio*, *Hyphomicrobium*, *Acidaminococcus*, *Bosea*, *Sutterella*, *Coprococcus*, *Pseudomonas*, *Methylophilus*, *Methylotenera*, *Blautia*, and *Faecalibacterium*. These emerging genera were more clustering and more abundant than the other three groups of samples. The relative abundance of other genera except for TOP 40 from S1 to S4 increased from 18.0% to 26.5%. From the mean relative abundance in all samples, the results showed that *Undicbacterium* and *Acidovorax* acted as the dominant genera in Yongding River when the water flow was reduced. With flow gradient restoration, each genus gradually prosperity, showing positive ecological response at the genus level.

### Response of bacterial community to water quality indicators

**Figure 5:**
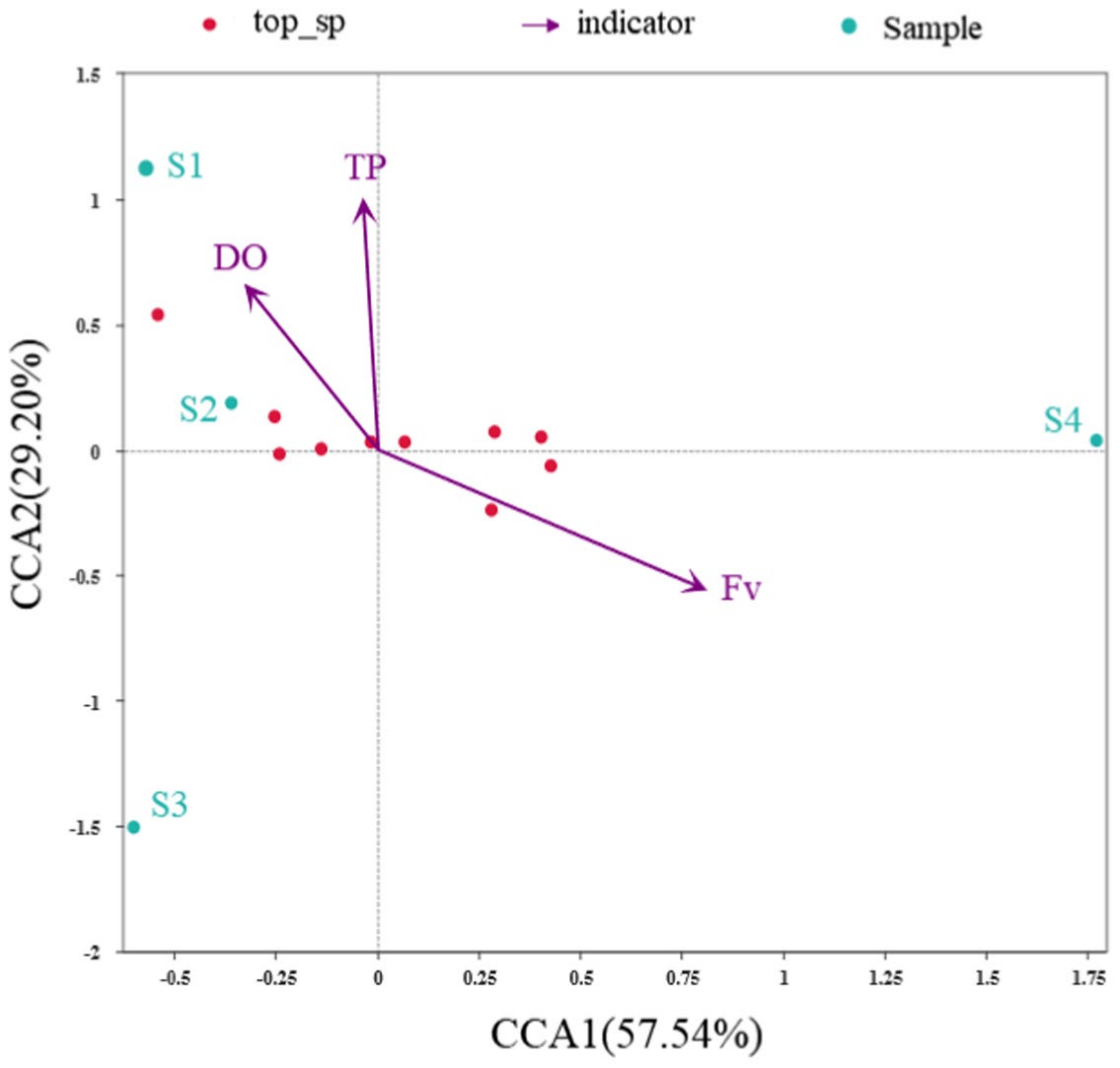
CCA Plot of the relationship between environmental indicators and the microbial community at the genus level (Note: There were too many indicators associated with the microbial community, many of which were autocorrelated. Therefore, only the environmental indicators that have a greater impact on the microbial community were retained).

CCA was conducted to determine the primary environmental variables associated with changes in the bacterial community structure (Figure 5). The first two axes explain up to 57.5% of CCA1 and 29.2% of CCA2 of the total variation in the bacterial community structure, indicating that the selected environmental indicators drive the differences in bacterial community structures. All the environmental indicators investigated, Fv (*R*^2^ = 0.986, Pr = 0.125) obviously have the most significant influence on the bacterial community, and most variances can be explained by TP (*R*^2^ = 0.955, Pr = 0.167) and DO (*R*^2^ = 0.529691, Pr = 0.75). In the CCA ranking diagram, the arrows of Fv have the longest length, indicating that the correlation between Fv and community distribution and species distribution was the largest. In short, water flow has a greater impact on bacterial community diversity and is the main indicator affecting bacterial community changes in the simulated flume. It is further proved that flow restoration in gradient obtains a positive response of microbial community structure.

### Response of microbial community FAPROTAX functional prediction to flow in gradient

**Figure 6:**
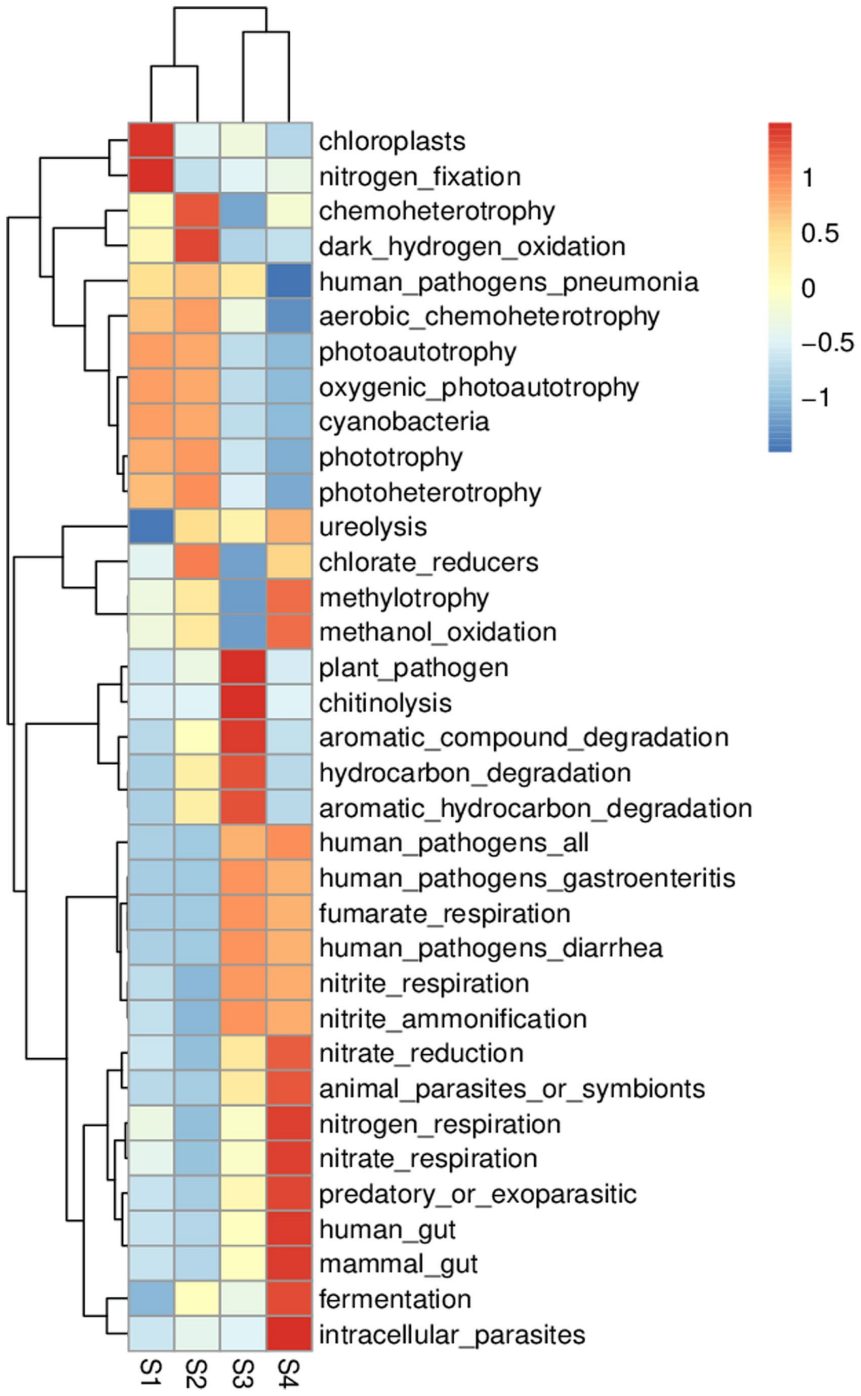
FAPROTAX analysis heat map (predicting the function of bacterial communities).

Community prediction analysis using FAPROTAX was conducted to determine the function of the observed bacterial community and ecological environment. In the four samples, with a flow gradient restoration, differences in community and ecological functions occurred (Figure 6). Based on the Prokaryotic Functional Annotation (PFA) database, more than 80 functional classifications were obtained, such as element cycling, microbial autotrophy and heterotrophy, animal and plant pathogens, fermentation and nitrate respiration, etc. In these four samples, the microbial functions were mainly chemoheterotrophs (38.3%), nitrate reduction (3.6%), chloroplasts (3.2%) and nitrogen respiration (2.9%). The heat map shows the dynamics of bacterial and ecological functions at the four sampling points (Supplementary Figure S2). FAPROTAX function prediction showed that in the S1 stage, the diversity of the microbial community was low, the biological functions of chloroplasts and nitrogen fixation were dominant, and the community was in the early stage of water ecosystem restoration. In the S4 stage, the microbial community not only has basic functions but also has the functions of nitrate reduction, nitrogen respiration, nitrate respiration and fermentation to regulate the aquatic ecological environment. In short, the gradient boost of flow can accelerate the restoration of water ecosystem function.

## Discussion

In this study, we simulated a flow gradient restoration in the flow-reduced river reach, performed high-throughput sequencing of microbial communities to explore the response of bacterial communities to water flow, and examined the response of water quality index to water flow. The alpha diversity results of 16s rRNA high-throughput sequencing indicated that the bacterial community structure generally showed a positive ecological response to a flow gradient restoration. In aquatic ecosystems, microorganisms play an important role and are sensitive to changes in freshwater flow (Bunn and arthington, 2002; Poff et al., 2007). This study focuses on the effects of flow on biodiversity, community structure and composition. The alpha diversity index is a commonly used indicator to reflect the species abundance and diversity of microbial communities (Erős et al., 2020; Meng et al., 2021; Qin et al., 2021). From the perspective of the Alpha diversity index, the number of OTUs, Ace, Chao, Shanoon and Simpson indices of the bacterial community had significant differences with the restoration of the gradient of water flow. It further proves that the microbial ecosystem in flow-reduced river reaches are dysfunctional, and needs to regulate water flow to maintain the microbial ecosystem properly. In addition, the improvement of the alpha index of the bacterial community may be specifically attributed to the fact that the increase in water flow accelerates the material exchange between the bacterial community and the water column, which promotes microbial metabolic proliferation (Tsai et al., 2013; Algarte et al., 2017). Microbial community structure showed positive ecological response with a flow gradient restoration, and the richness and diversity of the community were restored steadily. The alpha diversity index can be a useful tool for researchers to assess the microbial ecosystem in flow-reduced river reaches.

At the phylum level, the dominant phyla of the bacterial community in the simulated flume were *Proteobacteria*, *Firmicutes, Bacteroidetes* and *Cyanobacteria*. The sequencing results are similar with the results of bacterial community structure in many freshwater rivers, and the overall structure is consistent with the structural composition of typical bacterial communities in freshwater rivers (Zhang W. et al., 2022; Zhang Z. et al., 2022; Obieze et al., 2022). *Proteobacteria* have excellent adaptability and survival ability (Tang et al., 2021; Feng et al., 2022), can remove nitrogen and phosphorus while utilizing organic matter (Chen et al., 2021; Ren et al., 2022), and are widely distributed in aquatic ecosystems (Stevens et al., 2005; Ghate et al., 2021; Wang et al., 2022). Most of the common denitrifying bacteria in sewage treatment belong to *Proteobacteria*, which is an important bacterial phylum for removing excess nitrogen from aquatic ecosystems (Yu et al., 2020; Zya et al., 2021). Furthermore, *Firmicutes* have excellent cell wall strength, which also makes them relatively abundant under extreme conditions (Alalawy et al., 2021; Yin et al., 2021). In this study, *Proteobacteria* dominated the community structure (78.0%), the second dominant phylum was *Firmicutes* (6.4%), and the second dominant phylum in conventional rivers was *Bacteroidetes* or *Actinobacteria* (Newton et al., 2011; Liu et al., 2012; Qian et al., 2021). Another study found that the relative abundance of *Firmicutes* in freshwater aquatic ecosystems is generally less than 1.0%, and the relatively high abundance may be caused by animal fecal contamination (Vadde et al., 2019). With a flow gradient restoration, the relative abundance of *Proteobacteria* began to decrease from 78.0% to 75.5%. Compared to S1, new bacterial phyla began to be detected in S4, such as *Bdellovibrionota, Campylobacterota,* and *Myxococcota,* etc. We hypothesize that the increased water flow improved the water quality conditions. That is why new bacterial phyla or phyla with low relative abundance have been identified. In short, in the early stage of aquatic ecosystem restoration, *Proteobacteria* and *Firmicutes* with strong adaptability and survivability dominated. With the improvement of water flow and the gradual restoration of aquatic ecosystems, the overall structure of bacterial phyla tends to be diverse and comprehensive.

At the genus level, the genera with the highest relative abundance at each sampling point were *Undibacterium* (22.4%) and *Acidovorax* (19.1%), both belonging to *Gammaproteobacteria*. *Undibacterium* and *Acidovarax* are commonly used in municipal sewage treatment plants to remove carbon, nitrogen, phosphorus, sulfur and other elements in pollutants from water bodies in the form of microbial biomass (Qiu D. et al., 2021). It shows that the water quality conditions of the water body drained from the river were in a state of eutrophication. From the cluster heat map of bacteria and genus, the sampled genera clustered together, and with a flow gradient restoration, the clustering of genera was changed significantly. Through CCA analysis, it was determined that the main environmental variable related to the change of bacterial community structure was water flow, and the positive response of the bacterial community to water flow also promoted the positive response of other environmental water quality indices.

Many studies have shown that water flow, regarded as the main variable (Arthington et al., 2006; Poff and Zimmerman, 2010), underpins the basic functioning of river ecosystems. River metabolism can comprehensively reflect the photosynthesis of the primary and the respiration of the oxygen consumers of the ecosystem and is closely related to the hydrology, water quality, meteorology and other factors of the river basin. Therefore, the river metabolism can comprehensively reflect the function of the river ecosystem (Fellows et al., 2006). The river ecosystem (GPP and ER) is showing a positive ecological response to the restoration of flow. A variety of water quality indexes such as pH, DO, TN, TP TS, etc. in the water body can respond to the reduction of water flow (Colangelo, 2007; Staley et al., 2015; Joshi et al., 2017). However, studies on the responses of environmental water quality index to increases in water flow have been not comprehensive. In the cluster heat map, it was found that the relative abundance of *Hydrogenophaga* at the S2 sampling point was higher under its action, and the water pH was improved. The increase of Chla content is related to the eutrophication of the water body. The excessive organic matter in the water body leads to the growth of green algae, thereby increasing the Chla content in the water body (Zou et al., 2020; Qiu Q. et al., 2021). This also explains the reduction in DO below, as the growth of algae consumes DO in the water (Misra et al., 2011). However, NO_3_^−^ has a significant negative linear correlation with water flow, which is related to the *Proteobacteria* flora that can denitrify as mentioned above. In addition, TN, TP, TS, NO_2_^−^, NH₄^+^ and COD, excepted for DO, showed a negative correlation trend with water flow. The reduction of these indicators is related to the physical conditions of water flow on the one hand, and the metabolic decomposition of the microbial community on the other hand. The reduction in these water quality indicators also verifies that the increase in alpha diversity of the microbial community in the previous section is due to the acceleration of microbial proliferation and metabolism by the increase in water flow. To sum up, with a flow gradient restoration, the environmental function factors showed a relatively positive ecological response, and the water quality situation was improved.

With a flow gradient restoration, the microbial community structure and water quality conditions continue to improve. The microbial community not only has the function of basic element circulation but also has the functions of nitrate reduction, nitrogen respiration and fermentation to regulate the aquatic ecological environment. In short, from the perspective of microbial geophysicochemical element cycling, microbial community functions also have excellent ecological responses to water flow.

## Conclusion

In this study, we simulated the ecological response of environmental water quality index with a flow gradient restoration in the flow-reduced river reach and analyzed the response of the microbial community structure in the simulated flume by high-throughput sequencing technology based on 16 s rRNA. The results showed that the functional indicators GPP and ER, and the environmental water quality index pH, Chla, TN, TP, TS, NO_3_^−^, NO_2_^−^, NH₄^+^ and COD of aquatic ecosystems all exhibited positive ecological responses to water flow, except for DO. In the early stage of water flow restoration, the bacterial community diversity in the water environment was relatively high. The dominant bacterial phyla at the phylum level were *Proteobacteria, Firmicutes, Bacteroidetes* and *Cyanobacteria*. The dominant bacterial genera were *Undibacterium* and *Acidovarax*. These genera actively responded to changes in water flow and synergized with water flow to affect other environmental water quality indexes, promoting the improvement of water quality conditions. CCA analysis indicated that water flow, DO and TP were the main influencing factors for changes in bacterial community structure. The FAPROTAX function predicts that in the early stage of ecosystem restoration, bacterial communities first focus on basic biological functions, and then restore or adjust water environmental conditions through other microbial functions. In conclusion, this study revealed that water flow is the main indicator for the restoration of flow-reduced river reach, and studied the composition of bacterial communities and the ecological response of environmental functional factors and bacterial community structure to water flow. It lays a theoretical foundation for the ecological restoration of the flow-reduced river reach.

## Data availability statement

The data analyzed in this study is subject to the following licenses/restrictions: Restrictions apply to the availability of these data, which were used under license for this study. Data are available from the Weihuang with the permission of the Ministry of Water Resources of China. Requests to access these datasets should be directed to Weihuang huangwei@iwhr.com.

## Author contributions

WH: conceptuailization, investigation, writing-original draft, data curation, software, and vailidation. ZW and XL: investigation and data curation. DZ: conceptuailization, investigation, writing—original draft, and writing—review and editing. YW: conceptualization, funding acquisition, supervision, and project administration. LW: writing—original draft and writing—review and editing. All authors contributed to the article and approved the submitted version.

## Funding

This work was jointly supported by the National Natural Science Foundation of China (grant number 51879278), the IWHR Research & Development Support Program (grant number WE0145B052021), and the Open Research Fund of State Key Laboratory of Simulation and Regulation of Water Cycle in River Basin (China Institute of Water Resources and Hydropower Research; grant number IWHR-SKL-KF202107).

## Conflict of interest

The authors declare that the research was conducted in the absence of any commercial or financial relationships that could be construed as a potential conflict of interest.

## Publisher’s note

All claims expressed in this article are solely those of the authors and do not necessarily represent those of their affiliated organizations, or those of the publisher, the editors and the reviewers. Any product that may be evaluated in this article, or claim that may be made by its manufacturer, is not guaranteed or endorsed by the publisher.

## Footnotes

^1^ https://magic.novogene.com

^2^ http://www.drive5.com/uparse/

^3^ http://www.arb-silva.de/

^4^ http://www.drive5.com/muscle/
