# Supplementary material for: The microbial community and functional indicators response to flow restoration in gradient in a simulated water flume
Source: Front Microbiol. 2022 Nov 17;13:1051375. doi: 10.3389/fmicb.2022.1051375 (PMC9712197; doi:10.3389/fmicb.2022.1051375)
Supplement: Supplementary file 1 [file Data_Sheet_1.doc]

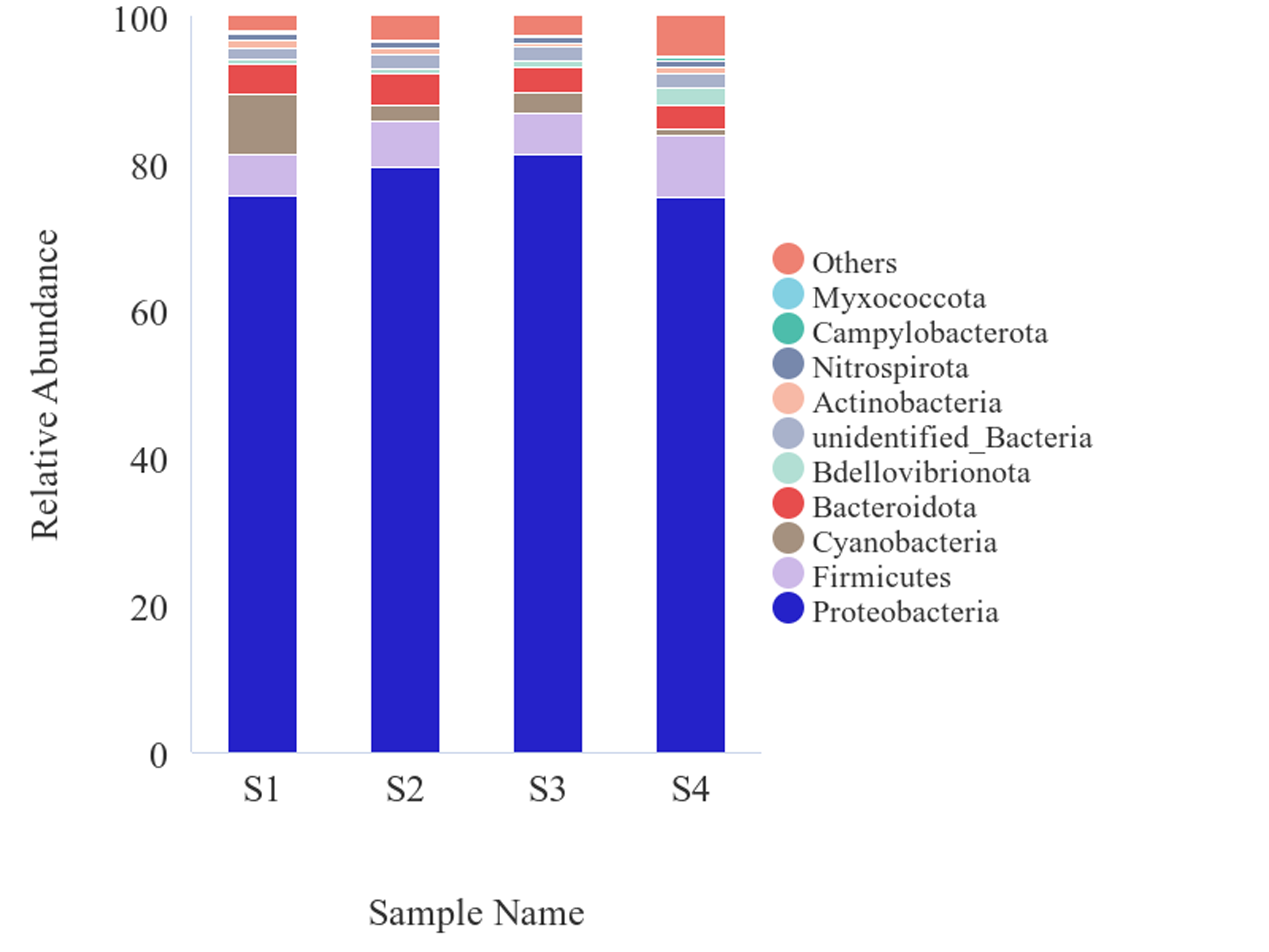


Fig. S1 Plots of relative abundance at each sampling site at the genus level.

####
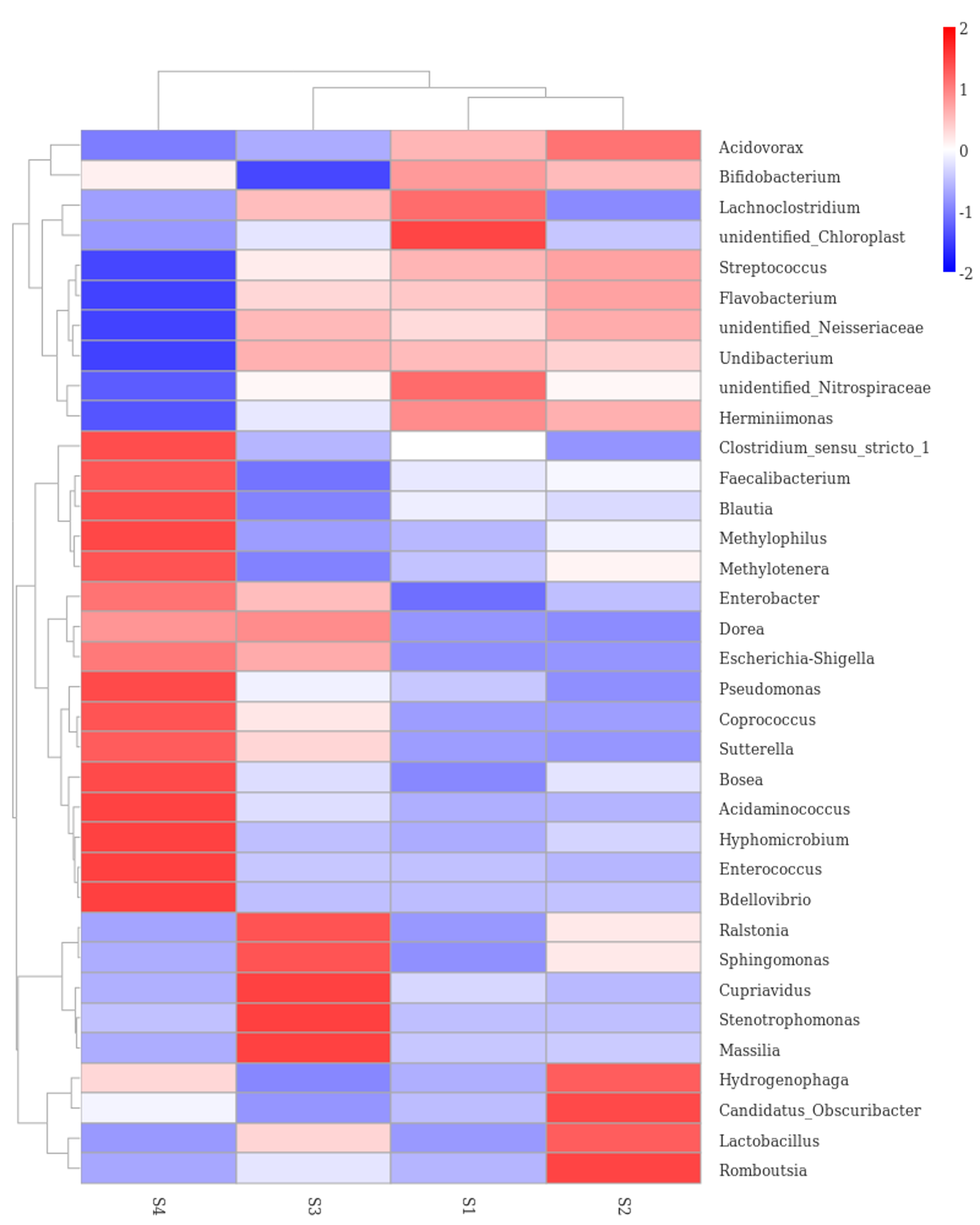


#### Fig. S2 Genus clustering heatmap (The dominant bacterial TAXA at the genus level)

#### **
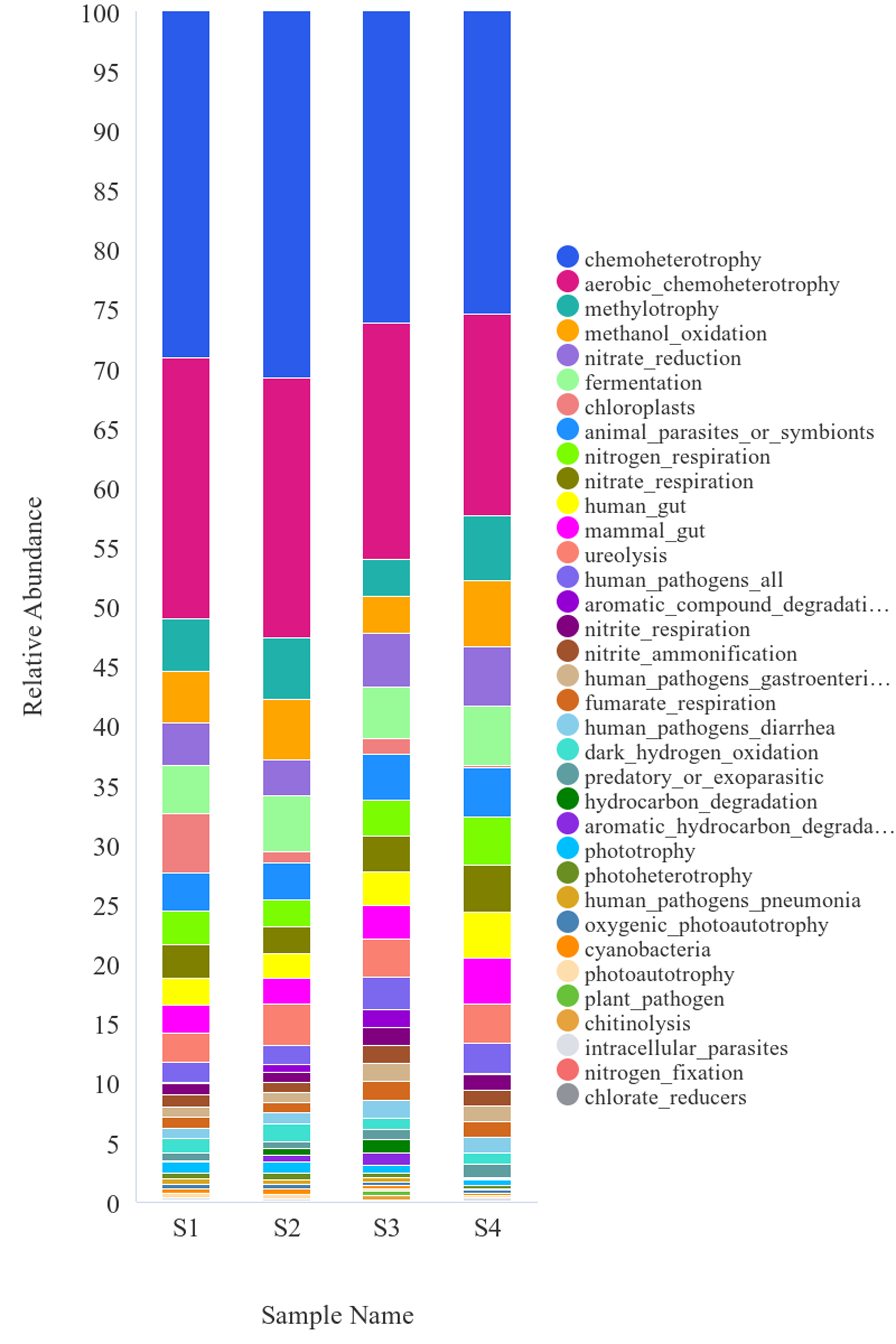
**Fig. S3 FAPROTAX - Relative abundance column chart
